# Phosphomolybdic acid-induced synthesis of self-incorporated flexible Ni(OH)_2_ nanosheets with enhanced photoreactivity

**DOI:** 10.1039/d5ra04699h

**Published:** 2025-09-12

**Authors:** Keshif Kazmi, Bilal Akrm, Muhammad Umer Fayaz, Amna Munsaf, Mudussar Ali, Ashfaq Ahmad Khan

**Affiliations:** a Department of Chemistry, Women University of Azad Jammu and Kashmir Bagh, AJ&K 12500 Pakistan bai-l16@mails.tsinghua.org.cn; b Department of Materials Science and Engineering, Southern University of Science and Technology Shenzhen Guangdong 518055 China; c Institute for Advanced Studies, Shenzhen University Shenzhen China

## Abstract

The rational design of flexible and crumpled nanosheet hybrid materials integrating heteropoly acids (HPAs) and metal hydroxides offers great potential for advanced photocatalytic applications. However, conventional syntheses often require complex ligands or templates. Here, we report a ligand-free solvothermal approach to fabricate a structurally unique phosphomolybdic acid (PMA)-nickel hydroxide Ni(OH)_2_ hybrid using a simple solvent system, avoiding long-chain stabilizers. The incorporation of PMA into the reaction system induces a flexible, crumpled nanosheet morphology, as confirmed by electron microscopy, while preserving the Keggin structure of PMA. This synergistic integration endows the hybrid with exceptional photocatalytic activity, achieving ∼97% degradation of methylene blue (MB) under light illumination using a 20 W power LED, outperforming pure Ni(OH)_2_ (74%) due to enhanced charge separation. Moreover, the hybrid exhibits outstanding recyclability over four cycles without performance loss, attributed to its robust structural integrity. Beyond photocatalysis, the flexible yet stable architecture of PMA-Ni(OH)_2_ suggests potential for energy storage or sensing applications. This work demonstrates a facile, scalable route to POM-based hybrids and highlights their multifunctional versatility through tailored nanoarchitectonics.

## Abstract 2

The incorporation of phosphomolybdic acid (PMA) induces crumpling and flexibility in Ni(OH)_2_ nanosheet structure while preserving its intrinsic Keggin structure.

## Graphical abstract

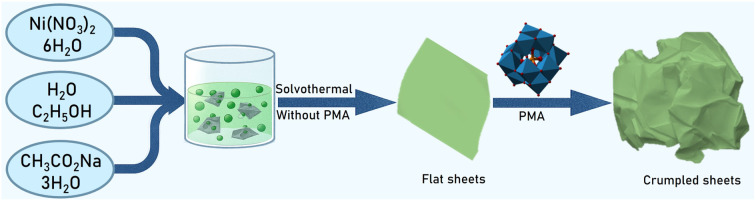

## Introduction

The development of high-performance nanomaterials for energy and environmental applications demands innovative solutions to overcome inherent material limitations.^1–4^ Nickel hydroxide (Ni(OH)_2_) shows great promise for energy storage and photocatalysis due to its low cost and environmental compatibility.^5,6^ However, its widespread adoption has been hindered by fundamental challenges including poor conductivity, limited surface area, and rigid structure.^7^ Recent advances suggest that nanostructuring, doping, hybridization with other functional materials and engineered flexibility could address these limitations.^8–10^ Among nanostructures, flexible nanosheets (NSs) offer enhanced surface area, mechanical durability, and adaptability for next-generation devices.^11–16^ However, creating such flexible inorganic nanostructures remains exceptionally challenging due to their intrinsic lattice rigidity and the complex processing.^17–20^ Unlike organic materials, which can exhibit flexibility through molecular rearrangements and weak intermolecular forces, inorganic NSs often lack the mechanisms for energy dissipation and deformation. Achieving flexibility in these materials requires precise control over their thickness, defect engineering, and sometimes the introduction of organic–inorganic hybrid structures.^21–23^ In this context, we demonstrate a breakthrough approach using heteropolyacids (HPAs) to simultaneously induce flexibility and enhance functionality in Ni(OH)_2_ NSs. This simple yet effective strategy bypasses conventional structural constraints, creating crumpled, free-standing architectures with improved performance characteristics, thereby opening new possibilities for advanced material design.

HPAs particularly phosphomolybdic acid (PMA) emerge as transformative building blocks. HPAs are not just ordinary metal-oxide clusters; their unique combination of structural versatility, tunable redox activity, and multiple binding sites enables them to fundamentally alter nanomaterial growth behavior.^24–27^ When co-assembled with inorganic materials like Ni(OH)_2_, HPAs disrupt conventional crystallization pathways through noncovalent interactions, leading to unprecedented architectures.^28–33^ Unlike rigid heterostructure formed by traditional methods, HPA-induced assemblies create intrinsically flexible hybrids where the HPA clusters serve as molecular “hinges” between inorganic domains. This explains our innovative use of PMA to simultaneously induce flexibility and enhance functionality in Ni(OH)_2_ NSs. The resulting all-inorganic materials combine the best of both worlds: the stability of metal hydroxides with the tunable electronic properties of polyoxometalates^34^ creating a new platform for advanced energy and environmental applications.

This study presents a novel synthesis strategy for fabricating flexible Ni(OH)_2_ NSs Our approach uniquely enables incorporation of PMA into the synthesis process enables the formation of flexible NSs with enhanced structural integrity and photoreactivity. The self-incorporation of catalytically active PMA species into the Ni(OH)_2_ lattice, introduces additional energy levels within the bandgap, thereby improving the material's light absorption and charge carrier dynamics. The resulting Ni(OH)_2_ NSs exhibit remarkable flexibility, high surface area, and excellent photocatalytic performance under visible light irradiation.

## Experimental section

A 0.29 g of nickel nitrate hexahydrate (Ni(NO_3_)_2_·6H_2_O) was weighed and dissolved in 0.7 mL of ultrapure water and 10 mL of ethanol. Next, 0.8 g of sodium acetate trihydrate (CH_3_COONa·3H_2_O) was added to the mixture, followed by the introduction of 0.05 g of phosphomolybdic acid (PMA), where the mixture was stirred for 10 minutes. The resulting homogeneous mixture is then transferred to the autoclave, which was sealed and placed in an oven at 180 °C for 12 hours. The resulting powder product was collected, washed with distilled water and ethanol using centrifugation at 10 000 rpm for 5 minutes. The obtained product was dried in a heating oven at 80 °C for 24 hours and then subjected to characterization. Control experiments were conducted under same experimental conditions without PMA and sodium acetate.

## Characterization

XRD patterns of the samples were measured on a Bruker D8 Advance X-ray diffractometer using Cu Kα radiation (*λ* = 1.5418 Å). XPS signals were collected by a Thermo Fisher ESCALAB 250Xi spectrometer applying monochromatic Al Kα X-ray sources (1486.6 eV) at 2.0 kV and 20 mA. UV-vis absorption spectra were obtained by a Shimadzu UV 3600 spectrometer. HRTEM, dark-field STEM, as well as EDX element mapping tests were performed on a Field Electron and Ion Company (FEI) Tecnai G2 F20 S-Twin microscope at 200 kV. FT-IR spectra were recorded by using a PerkinElmer FT-IR spectrophotometer. Brunauer–Emmett–Teller (BET) surface area and pore size distribution were performed using a Quadrasorb SI-MP instrument. The pore size distribution was calculated from the BJH method.

## Photocatalytic degradation of dye

The photocatalytic performance of the PMA-Ni(OH)_2_ flexible NSs was evaluated by assessing the degradation of methylene blue (MB) dye under UV light illumination. For typical photocatalysis experiment, 5 parts per million (ppm) solution of methylene blue was prepared. A 10 mL aliquot of this solution was then mixed with 0.01 g of the PMA-Ni(OH)_2_ nanocatalyst. The reaction mixture was first stirred in dark for 30 minutes to establish adsorption desorption equilibrium. After dark stirring the mixture was agitated under UV light irradiation using UV-LED source (25 W). The photodegradation performance was monitored by taking absorbance of the methylene blue solution using UV-vis spectrophotometer. The catalyst stability was assessed through recycling experiments. The same experiment was repeated using pure Ni(OH)_2_ NSs.

## Results and discussion

**Fig. 1:**
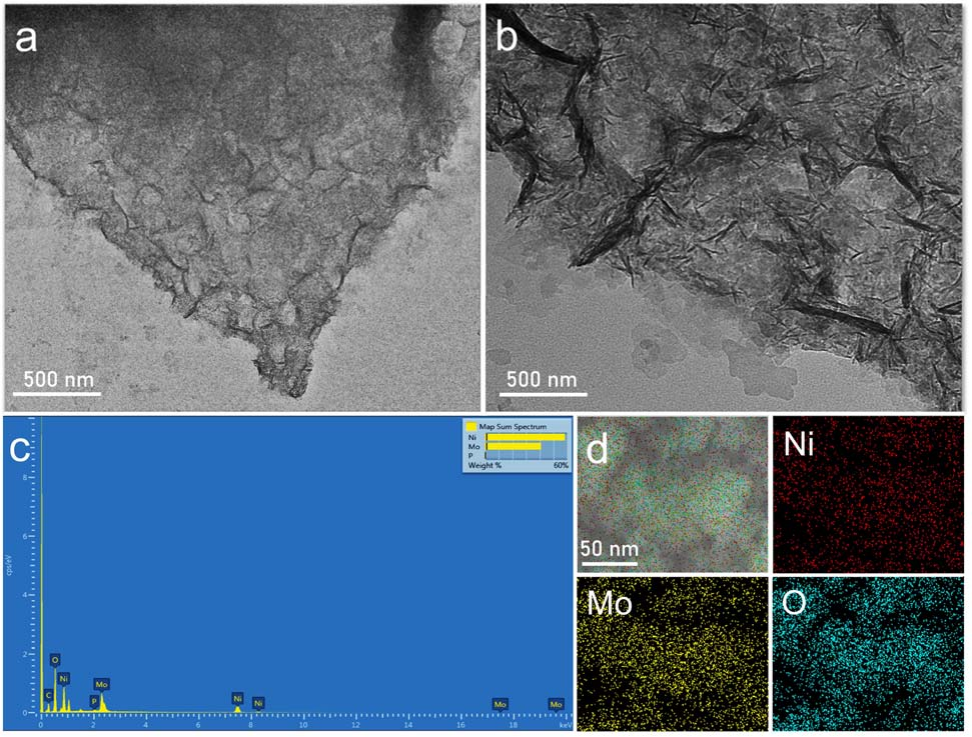
(a and b) TEM images at different magnifications, (c) EDX spectrum and (d) Elemental mapping through EDX of PMA-Ni(OH)_2_ flexible nanosheets.

The TEM images (Fig. 1a and b) demonstrate the two-dimensional sheet-like morphology of PMA-Ni(OH)_2_ flexible NSs, which exhibit enlarged lateral dimensions. The extended sheets undergo folding at multiple length scales, yielding a crumpled structure. The observed buckling and wrinkling further corroborate the flexibility of these NSs. Elemental composition analysis was performed using energy-dispersive X-ray (EDX) spectroscopy coupled with SEM. The EDX spectrum (Fig. 1c) confirms the coexistence of Ni and Mo species, while elemental mapping (Fig. 1d) reveals their homogeneous distribution throughout the NSs. This uniformity suggests effective integration of both components into the crumpled morphology.

It is worth mentioning that our developed reaction system is simple and does not involve the use of any long chain capping ligands or surfactants that usually insulates the resulting material and block the exposed active surface sites.^35^

**Fig. 2:**
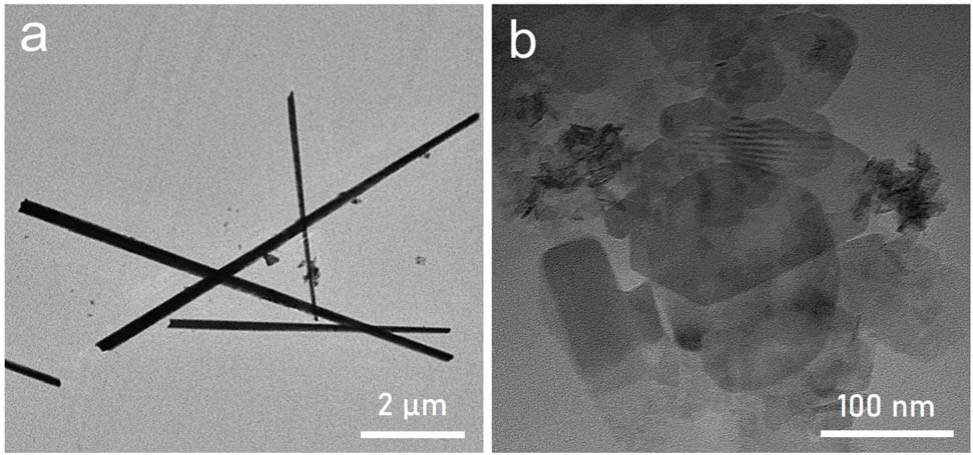
TEM image of product obtained (a) without sodium acetate and (b) without PMA.

Control experiments (without certain reagents) have been conducted to investigate the effect of each rection component on the determination of final crumpled morphology. In the absence of sodium acetate trihydrate one-dimensional microrods of PMA-Ni(OH)_2_ (Fig. 2a) have been obtained-whereas the absence of PMA in the system leads to the formation of free standing and relatively smaller NSs with no flexibility (Fig. 2b).

**Fig. 3:**
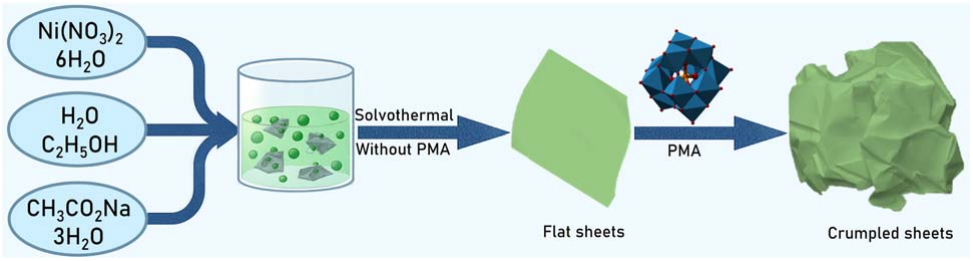
Schematic for the formation of PMA-Ni(OH)_2_ crumpled nanosheets.

Based on these observations it can be inferred that the sodium acetate not only act as reducing agent but also responsible for the morphology transition from 1D microrods to 2D free standing NSs. Furthermore, the catalytically active PMA clusters not only incorporate into the nanosheet structure to create a unique composite material but also impart structural flexibility to the resulting 2D architecture. The schematic of formation of crumpled PMA-Ni(OH)_2_ NSs is shown in Fig. 3. This change in morphology is likely due to PMA's influence on the growth process, promoting a more irregular, folded structure. This pictorial representation effectively highlights the impact of PMA on the NSs' morphology, emphasizing its role in creating a more complex and textured material.

**Fig. 4:**
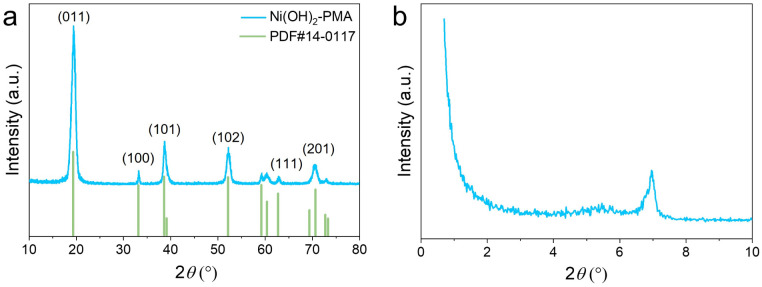
(a) XRD pattern and (b) small angle XRD pattern of PMA-Ni(OH)_2_ crumpled nanosheets.

The XRD pattern in Fig. 4a confirms the high crystallinity and phase purity of the synthesized PMA-Ni(OH)_2_ crumpled NSs. The well-defined diffraction peaks at 2*θ* values corresponding to the (001), (100), (101), (102), (111), and (201) planes match precisely with the reference pattern for Ni(OH)_2_ (JCPDS No. 14-0117), indicating the preservation of the crystalline structure after PMA incorporation. Notably, the XRD patterns of PMA-Ni(OH)_2_ NSs did not exhibit any additional peak characteristic of PMA, suggesting either homogeneous dispersion of PMA clusters within the Ni(OH)_2_ matrix at lower concentrations or amorphous incorporation of PMA species. These structural characterization results verify the successful synthesis of crystalline Ni(OH)_2_-PMA composite NSs while maintaining the host material's structural integrity. The small angle XRD pattern (Fig. 4b) reveals the appearance of a diffraction peak at 2*θ* ≈ 7° (*d* ∼12.6 Å) provides direct evidence for the ordered intercalation of PMA clusters within the Ni(OH)_2_ layered structure. This *d*-spacing value is significantly larger than that of pristine α-Ni(OH)_2_ (∼4.6–8 Å), confirming the successful interlayer incorporation of PMA anions. The observed spacing is consistent with the dimensions of Keggin-type PMA (∼10 Å diameter) surrounded by hydration spheres and charge-balancing cations, as previously reported for similar systems.^36,37^ The well-defined nature of this peak suggests that the PMA clusters are uniformly distributed between the hydroxide layers, creating a periodically ordered nanocomposite structure. This ordered intercalation likely contributes to the enhanced structural stability and unique electrochemical properties of the material, as demonstrated in previous studies of POM-intercalated layered hydroxides.^38^

**Fig. 5:**
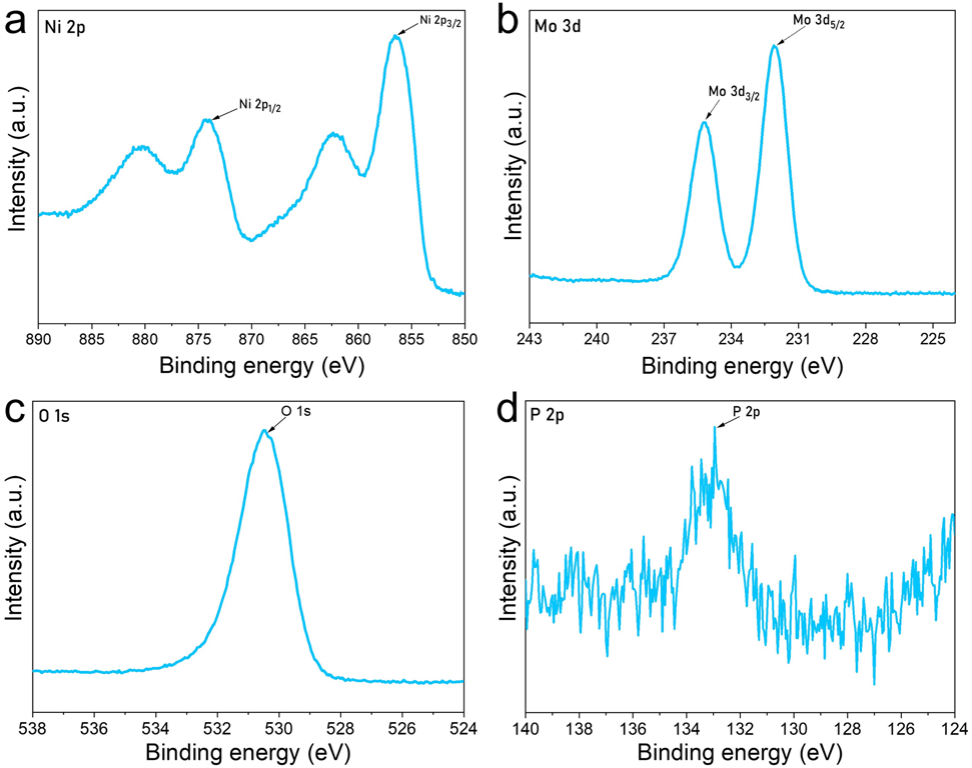
XPS spectrum of (a) Ni 2p, (b) Mo 3d, (c) O 1s and (d) P 2p moieties of PMA-Ni(OH)_2_ crumpled nanosheets.

The chemical states and interfacial interactions in the PMA-Ni(OH)_2_ crumpled NSs were elucidated through X-ray photoelectron spectroscopy (XPS). The Ni 2p spectrum (Fig. 5a) exhibited characteristic spin–orbit doublets at ∼856 eV (Ni 2p_3/2_) and ∼873 eV (Ni 2p_1/2_), consistent with Ni^2+^ in α-Ni(OH)_2_, as evidenced by the presence of shake-up satellite peaks (∼861 and 883 eV), which confirm the high-spin d8 electronic configuration of octahedrally coordinated Ni^2+^.^39^ A subtle yet discernible shift in the Ni 2p binding energies (BEs) of the hybrid, compared to pristine Ni(OH)_2_, suggests partial electron withdrawal from Ni centers, likely due to coordination with PMA oxygens (Ni–O–Mo linkages) or interfacial charge transfer. Concurrently, the Mo 3d spectrum (Fig. 5b) revealed peaks at ∼232 eV (Mo 3d_5/2_) and ∼236 eV (Mo 3d_3/2_), assigned to Mo^6+^ in the intact Keggin structure of PMA (PMo_12_O_40_^3−^).^40^ The absence of reduced Mo^5+^ species (typically observed at 230–231 eV) indicates PMA's structural stability during hybridization, while peak broadening implies electronic modulation at the PMA-Ni(OH)_2_ interface, possibly through Mo=O⋯H–O–Ni hydrogen bonding or d–p orbital hybridization.^41^ Collectively, these observations highlight electron redistribution from Ni(OH)_2_ to PMA, polarizing the interface and potentially enhancing redox activity. Such electronic synergism is critical for catalytic applications, as demonstrated in analogous Ni–POM systems where similar BE shifts correlate with improved oxygen evolution reaction kinetics.^42^

The O 1s spectrum (Fig. 5c) exhibited a dominant peak at 531.0 eV, which can be deconvoluted into two primary components: the lattice oxygen (O^2−^) in Ni(OH)_2_ (530.5–531.5 eV) and the terminal Mo=O bonds (531.0–532.0 eV) from the PMA Keggin structure.^9^ The comparable binding energies of these components resulted in peak overlap, suggesting strong interfacial interactions between Ni(OH)_2_ and PMA through potential Ni–O–Mo bridging bonds. The P 2p spectrum showed a broadened peak centered at 133.0 eV (Fig. 5d), characteristic of phosphorus in the PO_4_^3−^ unit of PMA, though the signal-to-noise ratio was reduced due to the low phosphorus content (1 : 12 P : Mo ratio in PMA) and potential surface inhomogeneity. The peak broadening may also indicate multiple chemical environments for phosphorus, possibly arising from partial protonation of PO_4_^3−^ groups or interactions with Ni(OH)_2_ surface hydroxyls.^43,44^ These observations collectively support the successful formation of a PMA-Ni(OH)_2_ hybrid material with strong interfacial electronic coupling.^45^

**Fig. 6:**
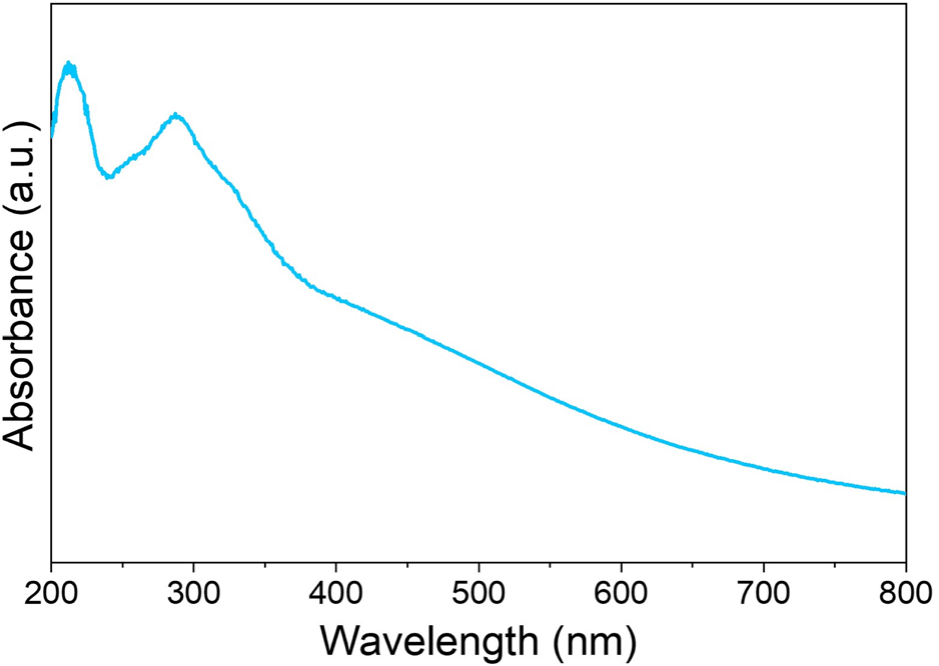
UV-visible spectrum of PMA-Ni(OH)_2_ flexible nanosheets.

The UV-visible spectrum (Fig. 6) of PMA-Ni(OH)_2_ crumpled NSs exhibits distinct optical characteristics that are crucial for its function as a visible-light photocatalyst. The sharp absorption peak observed around 300 nm can be attributed to the phosphomolybdic acid (PMA) component, specifically involving O^2−^ → Mo^6+^ interactions. This UV absorption indicates that PMA contributes primarily to the high-energy photoresponse of the hybrid. More importantly, the broad absorption spanning the visible region (400–800 nm) arises from d–d transitions of Ni^2+^ in an octahedral coordination environment within Ni(OH)_2_, along with potential contributions from defect states or interfacial charge transfer between PMA and Ni(OH)_2_. This extended visible absorption is particularly significant for photocatalytic applications, as it enables the material to harness a substantial portion of solar radiation.

**Fig. 7:**
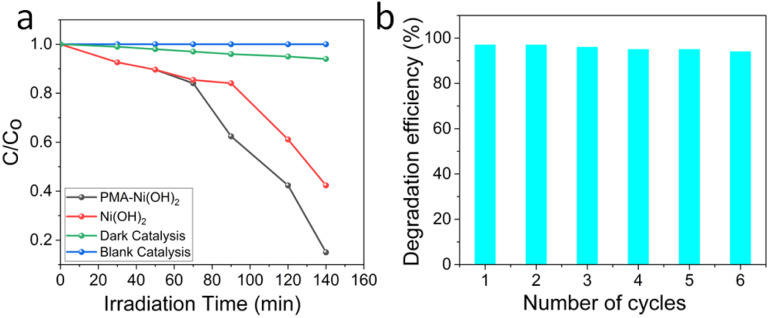
(a) Photodegradation of MB dyes under different conditions (b) stability test of PMA-Ni(OH)_2_ through recycling experiments.

The photocatalytic potential of newly designed PMA-Ni(OH)_2_ NSs was investigated using degradation of methylene blue a model textile dye waste.^46^ The hybrid PMA-Ni(OH)_2_ demonstrated remarkable photocatalytic efficiency (∼97%) under UV light irradiation, significantly outperforming pure Ni(OH)_2_ (74%) and the control experiments (no catalyst/dark conditions) as shown in Fig. 7a. This enhancement can be attributed to several synergistic factors:

Polyoxometalates (POMs), such as PMA, are known for their electron-accepting properties. When coupled with Ni(OH)_2_, PMA acts as an electron sink, effectively suppressing the recombination of photogenerated electron–hole pairs (e^−^–h^+^). The Keggin structure of PMA facilitates multi-electron redox reactions, enhancing the interfacial charge transfer to adsorbed dye molecules. Pure Ni(OH)_2_ exhibits moderate activity (74%) due to its intrinsic semiconductor properties (bandgap ∼2.5–3.0 eV), which allow UV absorption but suffer from rapid charge recombination. In the hybrid system, Ni(OH)_2_ likely serves as the primary photoabsorber, while PMA mediates electron extraction, leading to prolonged carrier lifetimes and higher oxidative species (˙OH, O_2_˙^−^) generation. The absence of activity in the dark confirms the photocatalytic nature of the reaction, ruling out adsorption or thermal effects.

The stability of PMA-Ni(OH)_2_ further underscores its practical potential. Even after six cycles of centrifugation and reuse, the hybrid retains its catalytic efficiency (Fig. 7b). The robustness of the hybrid can be attributed to the strong interfacial coupling between PMA and Ni(OH)_2_, which prevents leaching and maintains redox-active sites. Mechanistically, we propose that PMA's multi-electron redox capability facilitates the stepwise reduction of molecular oxygen to O_2_˙^−^, while Ni(OH)_2_^+^ holes oxidize water or surface-bound MB directly.

**Fig. 8:**
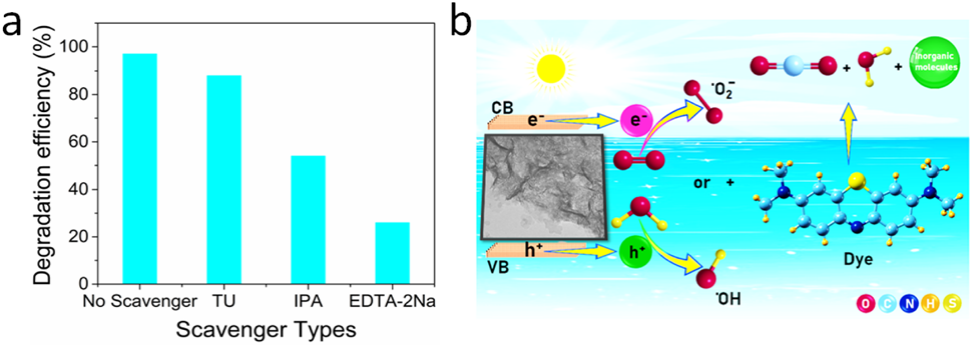
(a) Photocatalytic performance of PMA-Ni(OH)_2_ in the presence of various scavengers and (b) Pictorial representation of photodegradation mechanism.

The scavenging experiment was conducted to investigate the role of reactive species responsible for the photodegradation of dye. Sodium salt of ethylene diamine tetraacetate (EDTA-2Na), isopropyl alcohol (IPA), and thiourea (TU), have been used as different scavengers to investigate the role of active species such as holes (h^+^), hydroxyl radicals (O˙H), and superoxide anion radicals (O_2_˙^−^). In the absence of any type of scavengers, the photodegradation efficiency of the crumpled nanosheets catalyst was ∼97%. However, upon the addition of EDTA-2Na, the dye's degradation efficiency was reduced to 26%, and with IPA, it was reduced to 54% (Fig. 8a). Conversely, the addition of TU, which acts as a scavenger for O_2_˙^−^, resulted in only a slight decrease in dye degradation efficiency. This observation shows that h^+^ and O˙H radicals hold a significant role in degrading MB using PMA-Ni(OH)_2_ crumpled nanosheets as a photocatalyst. In contrast, thiourea scavenges superoxide radicals, which might be formed secondary to the primary hole-mediated reactions. Therefore, the observed significant decrease in photodegradation efficiency in the presence of EDTA-2Na suggests that hole-mediated reactions predominantly drive the degradation mechanism. The hole scavenger EDTA-2Na effectively competes with the MB molecules for available holes, inhibiting the degradation process and highlighting the significance of hole-mediated pathways in the photocatalytic degradation of MB. The mechanism of photocatalytic degradation was demonstated schematicaly in Fig. 8b.

## Conclusions

In summary, we have developed a novel one-step solvothermal method for synthesizing structurally and compositionally diverse polyoxometalate-based hybrids by co-assembling PMA clusters with Ni(OH)_2_. This approach enabled precise control over the size, morphology, and stability of the resulting structures. These hybrids demonstrated enhanced catalytic performance towards photodegradation of methylene blue dye and showed excellent recyclability.

## Conflicts of interest

There are no conflicts to declare.

## Data Availability

Data is available from the corresponding author upon a reasonable request.
